# Approaches to Assessment and Intervention with Children and Young People who have Experienced Child Sexual Abuse: A Review of Reviews

**DOI:** 10.1007/s40653-025-00702-4

**Published:** 2025-03-29

**Authors:** Lynne McPherson, Kathomi Gatwiri, Meaghan Vosz, Noel MacNamara, Janise Mitchell, Joe Tucci

**Affiliations:** 1 Centre for Children and Young People, Faculty of Health, Southern Cross University, Gold Coast, Australia; 2 Faculty of Health, Southern Cross University, Gold Coast, Australia; 3 Centre for Excellence in Therapeutic Care, Melbourne, Australia; 4 Centre for Excellence in Therapeutic Care, Australian Childhood Foundation, Melbourne, Australia; 5 Australian Childhood Foundation, Melbourne, Australia

**Keywords:** Child sexual abuse, Practice, Assessment, Intervention, Review of reviews

## Abstract

This paper focuses on understandings of Child Sexual Abuse (CSA) and best practice approaches in practice contexts to assessment and intervention with children who have experienced abuse. By collating data from existing reviews of literature or scoping reviews to formulate one overarching systematic scoping review, we provide a knowledge synthesis of practices in assessment and interventions into CSA. Utilising a two-phase scoping review method, the screening process resulted in twenty-two reviews for inclusion in this review. As a result of the analysis process, findings were identified across three broad themes: i) the limitations of current knowledge and official data about CSA and low rates of CSA prosecutions; ii) best practice in assessment and intervention; and iii) critical gaps in knowledge on CSA from diverse communities and those experiencing intersectional disadvantage. We conclude that whilst knowledge on CSA is continually emerging, there is limited consensus and significant challenges in the disclosure processes; poor skills in sensitive assessment and limited evidence about systemic responses, including low prosecution rates and poor integration of knowledge into practice; and promising interventions. There is also a critical gap in knowledge about CSA within racially and culturally minoritised groups, and other communities that report intersectional marginalisation.

## Introduction

Child sexual abuse is a prevalent social problem with high social and economic costs and serious, ongoing impacts on neurobiological development, psychosocial functioning and overall life trajectory. Worldwide estimates of child sexual abuse (CSA) prevalence are alarming, with an average of 18–20% of women and 8–10% of men reporting experiences of abuse (Pereda et al., 2009). More recently, the Australian Child Maltreatment Study (ACMS) has provided detailed information about CSA, finding “more than one in three girls” and “almost one in five boys experience child sexual abuse” (Matthews et al., 2023). According to *Recorded Crime in Australia* data (Australian Bureau of Statistics [ABS], 2019), more than 13,000 sexual assaults were perpetrated against children aged 10–17 years. These figures are considered an underestimation of CSA incidence in light of the barriers to children and young people’s disclosures and other systemic constraints to effective responses (Alaggia, Collin-Vezina & Lateef, 2019). In the context of disclosure, the Australia Royal Commission into Institutional Responses to Child Sexual Abuse, (2017) highlighted the difficulty victims of child sexual abuse may have in disclosing abuse. The inquiry heard from 7,981 survivors of child sexual abuse in 8,013 private sessions and was conducted over five years and produced its final report in 2017. This report found that 1 in 10 survivors who spoke to the Royal Commission never told anyone about their abuse (Royal Commission into Institutional Responses to Child Sexual Abuse, 2017) 

One central unresolved systemic issue is the lack of a shared definition and a rigorous conceptual model for understanding CSA (Mathews & Collins-Vezina, 2019). Different concepts have been used, including *child sexual abuse*, *child sexual assault*, *child sexual victimisation*, *child sexual exploitation*, *adverse sexual experiences*, and *unwanted sexual experiences*. This lack of a shared understanding of children’s diverse experiences can limit the capacity of researchers, clinicians, legislators, policymakers, and the wider community to quantify, treat, prevent, interrupt, and respond to CSA (Mathews & Collins-Vezina, 2019). This paper adopts the World Health Organisation (WHO) definition, which describes CSA as.

The involvement of a child in sexual activity that he or she does not fully comprehend is unable to give informed consent, or for which the child is not developmentally prepared and cannot give consent, or that violates the laws or social taboos of society. Child sexual abuse is evidenced by this activity between a child and an adult or another child who, by age or development, is in a relationship of responsibility, trust or power, the activity being intended to gratify or satisfy the needs of the other person (WHO, 1999, p.15).

Studies show that the critical combination of children’s vulnerability and adult-centric systems may contribute to children remaining in unsafe situations and at ongoing risk. Not all matters reported to authorities warrant investigation; however, there is a need to examine how the systems responding to child sexual abuse—including legal, health and child protection systems—facilitate safety and recovery for children and young people who have experienced child sexual abuse. In the context of known high rates of sexual abuse and definitional complexities in Australia, a review of evidence regarding CSA assessment and intervention was commissioned by the National Centre for Action on Child Sexual Abuse (NCACSA). In 2023, the review was refined to comprise a review of reviews as presented here. What follows is a detailed outline of the methodological process.

## Study Design and Methodology

The study was conducted across *two* distinct phases. A systematic scoping review was initiated in the first phase, with a plan to investigate empirical literature reporting assessment and intervention practices responding to child sexual abuse. This initial investigation employed strategies typically used in scoping reviews, including the development of clearly articulated research questions, using a transparent search approach with consistent keywords and inclusion and exclusion criteria (Pickering & Byrne, 2014). This is consistent with the goal of scoping reviews, which is to systematically investigate the scope of the available literature and “map relevant literature in a field of interest” (Arksey & O'Malley, 2005). Two research questions framed *phase one* of the project:

1. What is the current state of knowledge in the field of child sexual abuse, with reference to emerging practice issues?
2. What is known about best practice approaches to assessment and intervention with children who have experienced abuse?

### Search Strategy and Terms

The search strategy included the development of a search protocol with a qualified research librarian. The search included peer-reviewed scholarly articles written in English and published between 2012 and 2023. The period was selected to limit the results to recently published research in what is an evolving field of practice. Seven databases were searched: Academic Search Premier, CINAHL Plus with Full Text, Health Source: Nursing/Academic Edition, Master FILE Premier, MEDLINE with Full Text, APA Psych Articles and APA PsycInfo. Search terms included *child sexual abuse* OR *child sexual assault* OR *child sexual trauma*, AND children or child or young or youth or adolescent. AND *intervention* OR *best practice* OR *treatment* OR *therapeutic program* OR *assessment.*

### Phase One: Inclusion and Exclusion

In this phase, we included.

i) Studies with a clear objective designed to answer a specific research question
ii) Studies that collected analysed, and presented data
iii) Studies that used a rigorous methodology to identify multiple reviews that meet the quality of systematic or systematic scoping reviews
iv) Studies that i) addressed knowledge in the field of child sexual abuse, practice issues, assessment and/or ii) intervention approaches in the field of child sexual abuse.

We then excluded;

i) Studies that report on adult survivors of child sexual abuse
ii) Studies that did not address i) practice issues, assessment and/or ii) intervention approaches in the field of child sexual abuse.

### Results, Screening and Selecting Relevant Studies

The initial search of phase 1 yielded 4327 abstracts. After removing duplicates, two researchers independently screened the abstracts ensuring that the publication date range was as agreed, publications were written in English and focussed on the topic of child sexual abuse. This level resulted in 2883 articles being screened out with 1444 remaining.

Full texts of articles were then reviewed for eligibility based on the inclusion and exclusion criteria outlined earlier, as well as relevance to the research questions. This next level of screening resulted in 1142 articles being excluded, with 302 articles remaining. This process involved the six-member research team reading the **included 302** articles in order to confirm the selection.

Given the large volume of included papers (*n* = *302*), the research team considered a range of options, including further refinement of the research questions. Since the aim of the review was to consider and synthesise the current state of knowledge across a broad range of assessment and intervention approaches, the research team decided in favour of an approach that could efficiently and effectively include findings that reported on a broad range of practices. The breadth of articles available using the Cochrane method was seen to best address the research aims which was to scope or map the prevailing literature reporting on the state of knowledge broadly and assessment and intervention approaches in child sexual abuse. Cochrane review of reviews approach was then adopted for Phase 2 but still focused on practices that were implemented in response to the identification of CSA. The inclusion and exclusion criteria applied to the phase two review of reviews were as follows:

In this phase, we included.

(i) Studies with a clear objective designed to answer a specific research question
(ii) Systematic reviews, systematic scoping reviews, and literature reviews that collected analysed and presented data
(iii) Reviews that used a rigorous methodology to identify multiple reviews that meet the quality of systematic or systematic scoping reviews
(iv) Reviews that i) addressed knowledge, practice issues, assessment and/or ii) intervention approaches in the field of child sexual abuse.

We excluded;

(i) Single studies
(ii) Reviews that report on adult survivors of child sexual abuse
(iii) Reviews with a primary focus on preventing child sexual abuse were excluded.
(iv) Reviews that did not address i) addressed practice issues, assessment and/or ii) intervention approaches in the field of child sexual abuse.

By opting for a Cochrane review of reviews approach, we included 22 systematic or scoping reviews that were reported in more than 713 papers.

**Fig. 1:**
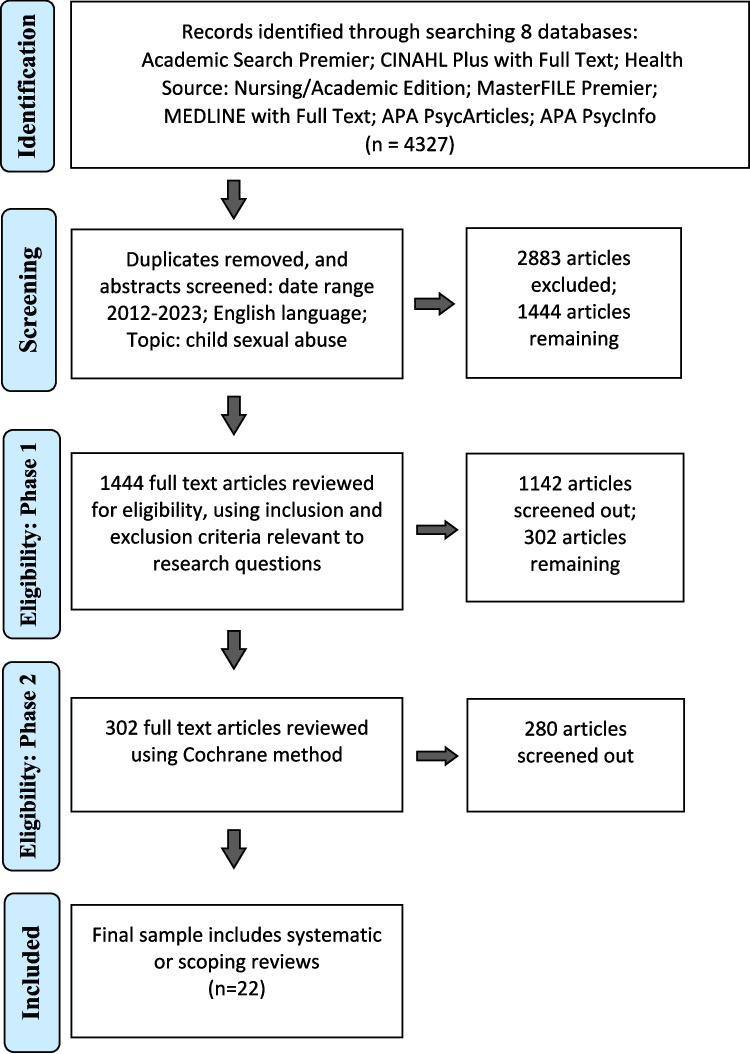
PRISMA Chart

Figure 1 uses the PRISMA process (Moher et al., 2009) to illustrate the identification, screening, and eligibility of articles included in the sample.

### Data Analysis

The findings of each of the 22 reviews were then analysed by the research team, who met on multiple occasions across four weeks to undertake intensive data analysis. The research team followed Braun and Clarke’s (2006) stages of thematic analysis, which facilitates the interpretation process of complex data, providing both depth and breadth of understanding to identify, analyse, and report patterns (themes) within the data (Braun & Clarke, 2021). This process involved firstly, organising and becoming familiar with the data, which included an immersion into the 22 reviews, which involved reading and re-reading the textual data and note-taking as a way to get a thorough understanding of the content. The second stage was making general sense of the data and generating initial codes. Here, the research team separately and together organised and labelled the “notes” into initial codes. At other times, this was done through paragraph-by-paragraph coding of the 22 texts. The next step was categorising the codes into themes by collating the codes in a a way that captured broader and more significant patterns in the data. The team engaged in an iterative and reflexive process throughout this phase, workshopping emergent themes with reference to the research questions. Next, we reviewed the themes that emerged and engaged in a process of interpretation. Here, we were able to split or combine themes if they were similar or explained similar phenomena. Finally, we agreed on how the analysis was to be presented and how each theme was to be described and supported by the data.

In summary, the 22 included reviews drawn from more than 713 articles in total (one review did not specify the number of included articles). Included reviews had a global focus (*n* = *17*) a focus on the USA *(n* = *2*), Australia (*n* = *1*), USA/Australia/Sweden (*n* = *1*) or USA/Canada/Australia (*n* = *1*).

A summary of the included publications is in Table 1.

**Table 1:** Findings: studies included in the review

Author	Year and location focus of review	Methodology & number of articles	Key Findings
Alaggia et al.	2019 Global focus	Systematic review of 33 articles	Disclosure is iterative, interactive process occuring within a relational context; contemporary models reflect social-ecological, person-in-environment orientation; age/gender influence disclosure; lack of a life-course perspective and barriers to disclosure outweigh facilitators (p. 260)
Broaddus-Shea et al.	2021 Global focus	Scoping review of 62 articles	Importance of respecting the child’s autonomy and wishes, ensuring privacy and confidentiality and making services appropriate and welcoming (p. 1)
Cross et al.	2003 USA focus	Meta-analysis of 21 articles	Most substantiated and founded cases do not lead to prosecution. Majority of prosecuted cases end in conviction, most by guilty plea. Charges less likely in CSA cases and more likely to be carried forward, but less likely to lead to incarceration other offenses (p. 334)
Gorissen et al.	2021 Global focus	Systematic review of 24 articles	Limited attention to components of online disclosure. Most studies focused on motivations for, and reactions to, online disclosure. Responses predominantly positive and negative reactions rare (p. 2)
Herbert and Bromfield	2016 USA, Australia and Sweden focus	Systematic review of 27 articles	Criminal justice outcomes of Child Advocacy Center (CAC) model well studied. Lack of research on the effect of the model on child and family outcomes and overreliance on measuring program outputs, rather than outcomes (p. 341)
Herbert and Bromfield	2019 Global focus	Systematic review of 63 articles	There is reasonable evidence to support the idea that multi-disciplinary teams (MDT) effectively improve criminal justice and mental health responses compared to standard practices (p. 214)
Kennedy and Prock	2018 Global focus	Systematic review of 123 articles	Self-blame, shame, and anticipatory stigma are barriers to CSA, SA, and IPV survivors’ disclosure and help-seeking. Abuse severity is related to self-blame and shame. Self-blame, shame, and negative social reactions are linked to poor outcomes. Self-blame, anticipatory stigma–related nondisclosure, and negative reactions predict sexual re-victimisation. Prior CSA exacerbates self-blame and stigma. Survivors from disadvantaged areas report higher stigmatisation/stigma (p. 521)
Kim et al.	2016 Global focus	Systematic review of 18 articles	Using a diverse set of treatment strategies improves adverse psychosocial sequelae of CSA. Cognitive behavioural therapy (CBT) is the most promising intervention (p. 597)
Macdonald et al.	2012 Global focus	Systematic review of 10 articles	Reporting of studies was poor, with significant weaknesses in quality. Evidence suggests CBT may positively impact the effects of CSA, including depression, posttraumatic stress and anxiety, but results generally modest (p. 2)
McTavish et al.	2021 Global focus	Systematic review of 24 articles	Limited evidence on the efficacy of psychosocial interventions for children, however, CBT with a trauma focus may have some impact on symptoms of PTSD. More research needed in lower-resource settings and countries
Mathews and Colin-Vézina	2019 Focus on the USA, Canada and Australia	Literature review of 6 prevalence articles	Law, policy and epidemiology define CSA, with variance in the approaches, the definition of acts, and the nature of consent. The result is multiple problems for research and knowledge formation, law, prevention, policy development and the shaping of social norms
McCalman et al.	2014 Australian Aboriginal and Torres Strait Islander focus	Systematic review of 23 articles	Program descriptions, measurement, and descriptive research were located, but no intervention studies. Insufficient evidence to confidently prescribe what works to effectively respond to sexual assault of Indigenous Australians (p. 1)
Nwogu et al.	2015 USA focus	Systematic review of 7 articles	Using CAC and the MDT approach in CSA investigation may positively increase non-offending caregivers’ satisfaction and prosecution rates of alleged sex offenders (p. 94)
Reitsema and Grietens	2016 Global focus	Exploratory review of 53 articles	Disclosure is a relational process, renegotiated by each interaction, evolving over time. The characteristics and reactions of the interaction partner are as critical as the behaviour and words of children themselves (p. 330)
Sánchez de Ribera et al.	2020 Global focus	Systematic review of 9 articles	AMSTAR-2 assists in a comprehensive appraisal of reviews. Overall finding of a ‘paucity of research’ (p.17)
Sawrikar and Katz	2017 Global focus drawing implications for Australia	Meta-analysis of 65 articles	CSA awareness in ethnic minority communities low in Australia. Barriers identified, and piloting culturally tailored programs recommended
Satapathy et al.	2016 global focus	Systematic reviewof 52 articles	Wide variety of methods and newer instruments were found, however many were outdated within the context of current child sexual abuse-related psychological trauma theories (p. 60)
Scoglio et al.	2021 global focus	Systematic review of 25 articles	Risk factors included: co-occurring maltreatment in the home, risky sexual behaviour, post-traumatic stress disorder, emotion dysregulation, and other maladaptive coping strategies. One protective factor identified: perceived parental care. Considerable variability in definitions and measurement, with limited generalisability (p. 41)
Tichelaar et al.	2020 global focus	Systematic review of 32articles	A range of psychotherapy techniques were identified—some demonstrated effectiveness. Some evidence that certain therapy approaches might be effective. A thorough comparison between studies was difficult because control groups and measured outcomes differed significantly (p. 1)
van Ham et al.	2020 global focus	Systematic review of 9 articles	Various aims and purposes for assessing nonverbal emotional signs were identified, with eight different, newly developed instruments. Insufficient information for analysis of reliability and validity. Need for one reliable, validated instrument (p. 12)
Vizard	2013 Global focus	Literature review included number unclear	Characteristics of victims are well known; those of juvenile perpetrators becoming recognised. Assessments should be delivered within a safeguarding context, with instruments used as adjuncts to full clinical assessment. Given high levels of psychiatric comorbidity, assessment, treatment, and other interventions should be undertaken by mental health-trained staff (p. 503)
Voogt et al.	2019 Global focus	Systematic review of 51 articles	Most experiments were conducted in USA with undergraduate students as participants, investigating cases with a male defendant and a female victim. Regarding victim credibility, approximately 60% of all measures were based on a single item, and 53% used materials not based on the child’s testimony. Credibility is measured using a variety of constructs including believability, honesty, truthfulness, suggestibility, accuracy, and reliability (p. 51)

## Limitations of Methodology

The decision to limit the review to a review of reviews was taken to enable the team to scope a large volume of studies, enabling a clear and rigorous synthesis within a manageable timeframe. That said, there may be several individual studies recently published that, whilst not included, may have added to the depth of the results of this present review. The research team formed the view that the breadth of manuscripts available using the Cochrane method would best address the research aim, while also retaining rigour and trustworthiness of the methodology.

It is also likely that this review has yet to identify all relevant literature due to the database searches, search criteria and the terms constructed by the research team. The research questions sought information about practice approaches; however, it is likely that the majority of practice approaches do not have program evaluations published in scholarly journals. The final limitation was that only reviews published in English and within the past ten years were analysed.

## Thematic Discussion

Following the thematic analysis process, findings were identified across three broad themes: i) the limitations of current knowledge and official data about CSA and low rates of CSA prosecutions; ii) best practices in assessment and intervention; and iii) system responses and gaps in knowledge to inform future research regarding the sexual abuse of children and young people and those experiencing intersectional disadvantage. Each major theme was classified into subthemes.

### Theme 1: Limited Current Knowledge and Official Data on CSA

Findings about the available scholarly evidence regarding CSA and practice approaches to assessment and intervention involved three critical flaws. This core theme includes and discusses three sub-themes: the limited consensus in the definition and conceptualisation of CSA, low rates of prosecution of CSA as a crime, and the apparent silence on literature responding to diversity in disability, culture and sexual orientation. Each of these are presented below.

#### i) Limited Consensus on CSA Definition and Conceptualisation

The absence of a shared language, which might enable a common understanding of the nature of CSA, has been raised as a point of concern repeatedly by researchers for decades. Experts suggest that concerted international attention to the problem cannot move forward without a shared conceptual definition (Finkelhor & Korbin, in Mathews & Collin-Vézina, 2019). Arguments are made that;

The term child sexual abuse has been defined differently, with epidemiology, policy and law using different approaches that construct child sexual abuse and the nature of consent. This variance produces multiple problems for research, policy, law, prevention and the shaping of social norms (Mathews & Collin-Vézina, 2019, p.139).

Mathews & Collin-Vézina (2019, pp. 135–6) have highlighted three areas of conceptual variance. Firstly, studies diverge in describing *the person engaging in CSA* and their relationship to the child. Some literature highlighted the role of trust and responsibility; other literature included sexual gratification as a core component of child sexual abuse. They also found definitional slippage regarding the *acts that constitute CSA*, with some research including contact abuse only in its scope while others included non-contact activities, such as online abuse and exploitation, or failed to define such acts. Finally, studies varied regarding *consent* as a critical component of the conceptualisation of CSA. Some studies contend that children cannot give informed consent; some argued that children are not developmentally prepared for the activity; while others neglected the issue of consent. The authors argued that the variance across these three dimensions leads to ambiguity in legislative, policy and practice frameworks and, inevitably, inconsistency across jurisdictions. Mathews & Collin-Vézina (2019, p. 146) proposed that conceptual coherence would improve understanding and responsiveness to CSA where such a model recognises its complexity and sensitivity.

Additionally, literature pointed to a gulf between self-reported experiences of child sexual abuse reported in research and rates of abuse recorded by official authorities such as child protection and the police. Studies estimated that self-reported abuse could be up to *30 times greater* than official reports to child protection or the police (Alaggia et al., 2019). These findings challenge official data capturing reports, investigations and substantiations of child sexual abuse, which could be linked to poor conceptualisations of CSA.

#### ii) Low Rates of Prosecution of Sex Offending Against Children

Reports to child protection authorities about possible sexual abuse may contain limited direct evidence of the abuse. There may be delayed and conflicted disclosure, non-verbal signs by the child, a lack of physical forensic evidence and no other witnesses to the abuse itself. There may be a retraction by the child of an initial disclosure. Cases may, therefore, be closed prematurely involving children and young people who are not being heard. In a meta-analysis of prosecutorial decisions concerning child abuse (including child sexual abuse), 21 studies conducted in the USA, found that charging rates varied considerably (Cross et al., 2003). An analysis of the wide variation in ‘prosecution rates’ also highlighted differences across state jurisdictions in policies and processes of assessing and responding to child abuse allegations. In states where there is engagement of multi-disciplinary teams and specialist professionals in CSA assessment and response, matters are more likely to be prosecuted. The authors concluded that:

Prosecution of child abuse is a specialised endeavour that requires skill in and commitment to child interviewing, gathering corroborative evidence and many other elements of investigation. It is likely that this skill is not evenly distributed, so some prosecutors ‘offices are likely to be better than others at developing cases that lead to charges (Cross et al., 2003, p. 335).

***This meta-analysis found that most substantiated and founded child abuse cases do not lead to prosecution and charges were less likely to be laid in CSA cases than other felonies.***

#### iii) Silence on Literature Responding to Diversity in Disability, Culture and Sexual Orientation

The apparent absence of literature addressing disability, culture, diversity, gender and sexual identity was notable among the studies sampled. There were no reviews in the included data set that focussed on the experiences and needs of First Nations children who had experienced child sexual abuse. Individual primary studies may have been undertaken; however, systematic reviews were not found. One exception was a systematic review regarding *services available* to Australian Aboriginal and Torres Strait Islander peoples following sexual assault (McCalman, Bridge, Whiteside, Bainbridge, Tsey & Jongen, 2014). The review included twenty-three publications reporting community-based and mainstream service responses for First Nations survivors and perpetrators. Whilst program descriptions and descriptive research were located, intervention and efficacy studies were not. Consequently, the authors could not conclude that evidence was available to effectively respond to the experience of sexual assault of Aboriginal and Torres Strait Islander peoples in Australia (McCalman et al., 2014).

Another review found that the level of awareness of CSA within ethnic minority communities in Australia was likely to be low, with barriers to enhancing awareness including cultural constraints (Sawrikar & Katz, 2017). Another study reviewing psychometric tests and scales designed to assess children and young people who had experienced CSA revealed that of the fifty-two scales reviewed, most (95.23%) were developed in the USA (Satapathy et al., 2016). This study, based in Asia, found that some scales were translated into Asian languages. However, no scales were found that were developed in Asian countries. The authors suggested that:

The absence of any efforts in Asian countries is surprising, particularly in view of the alarming increase in reporting. The adaptation and translation of these scales in Asia also lacked strength in the process and methodology. … A good evaluation and careful assessment reflect an integrative process that gathers disparate pieces of information together to enhance clinicians’ understanding of various features of a CSA victim’s life. Importantly, communicating this to the victims or their caregivers appropriately and adequately enables them to view their lives more accurately and positively (Satapathy et al., 2016, p.71).

None of the reviews focused on the needs of children and young people living with a disability who had experienced CSA. Similarly, there were no reviews of research into the support required by children and young people who identified as gender and sexually diverse.

### Theme 2: Evidence Relating to Assessment and Intervention

Within this broad theme, five subthemes emerged with implications for assessment and intervention in child sexual abuse matters. There were (i) the processes of disclosure of CSA, (ii) sensitive approaches to assessment, (iii) promising practice interventions,

#### i) Processes of Disclosure of CSA

A review by Alaggia, Collin-Ve´zina & Lateef (2019) analysed thirty-three studies investigating children’s disclosure and suggested that disclosure is an ongoing process, not a one-off event, but an iterative and interactive process. Earlier disclosures are facilitated by creating an open dialogue in a relationship that counteracts the perpetrator’s silencing influence (Alaggia, Collin-Ve´zina & Lateef, 2019). Barriers and enablers to disclosure included a complex interplay of individual, familial, contextual and cultural issues, with age and gender predictive of delayed disclosure for younger children and adolescent boys. Younger children were more likely to demonstrate behaviours that suggest they may have experienced abuse rather than deliberately talking about it.

Barriers to CSA disclosure identified in the sample often included experiences of shame, self-blame, and fear. A key finding in one large-scale study examining more than 1737 case files involving child sexual abuse was that more than half of the cases were detected through accidental disclosure or eyewitness detection (Collings, Griffith and Kamalo, cited in Alaggia et al., 2019). Less than one-third were purposeful disclosures initiated by the child victim. Recent studies have focused on motivations to disclose online (Gorissen, van den Berg, Bijleveld, Catrien, Ruiter & Berenblum, 2021). In their review of twenty-four studies, Gorissen et al. (2021) found that those who disclosed abuse online often did so to seek support, clarification and validation, to unburden, seek justice and to document the abuse.

Rates of disclosure increased with age and were often delayed until adulthood (Collings, Griffith and Kamalo, cited in Alaggia et al., 2019). Whilst delay of verbal disclosure was viewed as common in children, it was noted that children and young people may show a range of non-verbal signs that can be assessed (van Ham, van Konijnenburg, Brilleslijper-Kater, Schepers, Daams, Teeuw, van Rijn & van der Lee, 2020). These authors systematically reviewed research investigating children’s nonverbal and emotional signs during investigative interviews for suspected CSA. They provided an overview of eight newly developed instruments used to assess non-verbal signs, noting that their review did not provide sufficient information for analysis of reliability or validity. They concluded that there is a need for further research examining the assessment of non-verbal emotional indicators in children and young people who are the subject of a child sexual abuse investigation (van Ham et al., 2020).

#### ii) Sensitive Approaches to Assessment

A review of sixty-two publications concerning the initial health assessments of children who had experienced child sexual abuse extrapolated principles for good practice in assessment (Broaddus-Shea, Scott, Reijnders & Amin, 2021). The authors examined the response preferences of children and young people who had been sexually abused, along with those of their carers and health workers, culminating in a proposed set of child-sensitive guiding principles for assessment practice. The review revealed the importance of responding to children and young people “in a manner that respects the child or adolescent’s autonomy and wishes, ensures privacy and confidentiality, and makes services and facilities appropriate and welcoming” (Broaddus-Shea et al., 2021, p.1). The authors concluded that their findings should inform evidence-based assessments in a trauma-informed and child-sensitive manner.

#### iii) Promising Practice Interventions

The literature related to effective intervention was very limited in scope. In general, therapeutic interventions that include arguably adult-centric concepts such as *treatment*, *recovery* or *symptom reduction* were the main focus of the limited research reported. As such, it was not possible to evaluate the broad set of strategies that meet the needs of children and young people who have experienced sexual abuse.

The current paper identified four reviews, reporting on a total of 84 studies, which examined aspects of interventions or therapeutic approaches to respond to child sexual abuse (Kim, Noh, & Kim, 2016; Macdonald, Higgins, Ramchandani, Valentine, Bronger, Klein, O’Daniel, Pickering, Rademaker, Richardson, & Taylor, 2012; McTavish, Santeso, Amin, Rejnders, Ali, Fitzpatrick, Lewisi, & MacMillan, 2021; Tichelaar et al., 2020). The major approaches to intervention or treatment responses to CSA identified in these reviews were Cognitive Behavioural Therapy (CBT), CBT with a trauma focus or using prolonged exposure, eye movement desensitisation and reprocessing (EMDR), family meetings, and individual and group psychotherapy (Kim et al., 2016; Macdonald et al., 2012; McTavish et al. 2021).

There was limited evidence demonstrating the effectiveness of any of the interventions. CBT and CBT with a trauma focus were reported to be the most widely used treatment approaches for the CSA field and regarded by one review as the most promising approaches (Kim et al., 2016). However, all of the reviews in this sample highlighted the poor quality of available evidence. Studies examining the effectiveness of these interventions were difficult to compare because of variable interventions and study designs. Methodological limitations also included serious concerns about risk of bias (McTavish et al., 2021).

A Cochrane Collaboration review of CBT interventions with children who had been sexually abused included ten studies using randomised controlled or quasi-randomised controlled trials and involving a total of 847 participants (Macdonald et al., 2012). It concluded that:

The reporting of the studies was poor and there appear to be significant weaknesses in study quality. The evidence suggests that CBT may have a positive impact on the effects of child sexual abuse, including depression, post-traumatic stress and anxiety, but the results were generally modest (Macdonald et al., 2012, p.2).

Similar findings were made by McTavish et al. (2021) in a systematic review of psychosocial CSA interventions, including child-only therapies and child and caregiver interventions. They concluded that CBT and manualised CBT with a trauma focus were promising interventions. The authors consequently made a conditional recommendation to the WHO guideline development group to endorse CBT with a trauma focus for sexually abused young people experiencing PTSD symptoms (McTavish et al., 2021). Other interventions in this review that showed promise yet needed further evidence were prolonged exposure, individual psychotherapy, and family therapy, which focussed on risk reduction. The authors cautioned that cultural relevance should be considered, in particular when adapting interventions from high-income countries into low and middle-income settings (McTavish et al., 2021).

### Theme 3: Service System Responses

The literature included in the current scoping review was limited in its reporting on systems responding to child sexual abuse. Reports did include a North American response system known as the Child Advocacy Centre (CAC) model, which has been in operation for 40 years in the USA (Herbert & Bromfield, 2016),

which includes a multi-disciplinary team intervention, was developed to provide a coordinated effort to investigate allegations of CSA and treat children while minimising trauma and maximising successful prosecution of alleged child sex offenders……Child Advocacy Centres are child-friendly, facility-based programs with agents from various disciplines functioning together to efficiently examine, prosecute and treat allegations of child abuse (Nwogu et al., 2015, p.93).

The CAC approach evolved to include a range of investigative, medical and therapeutic services in a single child-friendly location to support the child’s recovery from trauma (Herbert & Bromfield, 2016). In their systematic review of 27 studies reporting on the CAC model, Herbert and Bromfield (2016) found that most focused on criminal justice outcomes as opposed to children’s wellbeing. Consequently, there was limited evidence in relation to the CAC model for child trauma outcomes, even though a stated aim of the CAC model was to reduce systemic trauma associated with responses to child sexual abuse.

A more recent systematic review of multi-disciplinary team responses to child physical and sexual abuse examined 63 articles implementing a range of approaches, including the CAC model (Herbert & Bromfield, 2019). Evidence for the outcomes of multi-disciplinary team approaches was collated across five categories:

1. *criminal justice outcomes*
2. *mental health/support service referral and improvement in trauma symptoms*
3. *child protection outcome*
4. *satisfaction with the response, and*
5. *medical referral and improvement in medical symptoms (*Herbert & Bromfield, 2019*, p. 218).*

Findings suggested reasonable evidence supporting multi-disciplinary teams as a whole-of-system response to child sexual abuse to improve criminal justice *and* mental health responses, compared to standard agency practices.

A notable gap in the literature was the role of lived experience of CSA as a source of knowledge to shape practice, programs and policy, influencing system responses in a critical way. Exceptions were those articles reporting on the role and involvement of victims/survivors in the Australian Royal Commission into Institutional Child Sexual Abuse (Blunden, Giuntoli, Newton & Katz, 2021; Wright, Swain & McPhillips, 2017). Described as a “landmark public inquiry” (Wright et al., 2017, p.1), it commenced an investigation into each institution with testimony from victims/survivors. The Commission kept people informed about its work and instigated a wide-ranging research and policy agenda informed by their testimonies. A qualitative study completed as part of this agenda explored the supportive responses of institutions and aimed to inform best practice responses to institutional child sexual abuse (Blunden et al., 2021).

## Discussion

Consistent with the identified research questions for this review, this discussion focuses on the state of knowledge in the field of child sexual abuse along with emerging practice implications. Attention paid to discussing findings concerning assessment and intervention approaches highlights that incongruence between rates of self-reported abuse and official data raises questions about how service systems identify and respond to the sexual abuse of children and young people. 

The potentially serious implications for child victims suggest a better understanding of what helps children and young people raise concerns or disclose CSA. Advancing this knowledge may enable service systems to develop responses that enhance the possibility of early disclosure, allowing the potential for protection from further abuse. The findings of the current review indicate that children and young people need adults to be alert to their early attempts to break the secret of child sexual abuse.

The research suggests that the evidence supporting intervention approaches is consistently methodologically faulty. The most common goal of the interventions was the reduction in various symptoms, such as PTSD or high-risk behaviours (Tichelaar et al., 2020). Some approaches identified as holistic models of intervention sought to respond to and address the broad needs of and underlying trauma children who have experienced CSA suffer. However, these models have not generally been evaluated. A reasonable conclusion is that there can only be a low confidence level in the effectiveness of intervention approaches.

This review revealed critically low rates of official data reporting child sexual abuse. It found that how children attempt to communicate the abuse they have experienced may take place over time and may not be recounted in a clear and coherent verbal account of the details.

For children and young people who have endured the trauma of child sexual abuse, as Porges suggests, “Feeling safe is the treatment” (Porges in Mitchell, Tucci & Tronick, 2020, p.11).

These children (as survivors of trauma and abuse) have never had the opportunity to experience a prolonged period of safety consistent with their bodily needs for health, growth, and restoration (Porges, cited in Mitchell, Tucci & Tronick, 2020, p.13).

It was noted earlier that the official data suggests incredibly low reporting rates of child sexual abuse. From a low official report baseline, a mere 12% of reports result in a conviction. This raises questions about the fundamental requirement for recovery from sexual abuse, that of the safety of most children where reporting has not resulted in a conviction.

## Recommendations and Implications for Policy and Practice

- A common definition of CSA is needed, as well as an international consensus about what interventions to target, how they should be implemented, and the outcomes that should be expected from them. Lived experience is a valuable source of knowledge that can inform service design, evaluation, and policy (Khoo et al., 2015; Michell, 2015; Vosz, 2021; Zaffarese-Dippold, 2017). Investment in critical, participatory studies that elevate children's and young people's experiences is warranted to build the evidence for effective intervention.
- A need to bolster global knowledge by including and publishing studies from culturally and contextually diverse settings is evident. There can be no practice implications drawn that are culturally and contextually responsive in the absence of research conducted in various settings and across diverse populations. The CSA research literature appears to be predominantly white and Western. When it comes to tools for assessment, screening, and measurement of symptomology, it is almost exclusively North American.
- Exploring studies focussing on assessment and intervention also raises significant questions about how legal, child protection and health systems respond to child sexual abuse. Child sexual abuse is arguably a welfare, criminal/legal *and* health issue. Welfare responses would include psychosocial approaches and child-friendly environments. However, the dominant lens appears to be a medical model response to child sexual abuse (Broaddus-Shea et al., 2021). Future research examining best practices in assessment approaches could adopt a wider lens and consider multi-agency and multi-disciplinary assessment practices across disciplines and professional settings.
- Globally, there is little evidence about the assessment and intervention of CSA in ethnic minority communities (Sawrikar & Katz, 2017). In responding to this silence, researchers and practitioners should adopt a critical, intersectional approach to sexual abuse research and practice within First Nations, ethnic minorities, people with disabilities and people with other unique and diverse intersections. This approach enables individuals and systems to confront the “positional and situational inequalities” (Bernard, 2019, p. 193) that render minoritised children and young people victims to higher prevalence, “longer and harsher abuse” (Klebanov et al., 2024, p. 1296). Further work is therefore needed to build the evidence base about effective assessment and intervention practices that respond to the specific needs of racially and culturally minoritised groups as well as First Nations people in a culturally respectful and dignified way.
- Regarding the state of knowledge, conceptualisation differences concerning CSA suggest the need to develop an international consensus. Whilst different national specificities in the law may present as a constraint, an international consensus might enable a more accurate and consistent measure of prevalence within and between countries and provide the basis for a common language across areas of the law, including child protection, criminal prosecution, and civil litigation.

## Conclusion

This review aimed to source and synthesise the current state of knowledge in child sexual abuse to identify practice issues. Based on these findings, there are several practice, policy, and further research considerations. An overall finding is that a whole-service system analysis is required. This analysis should critically examine how children and young people can be protected from child sexual abuse and, where sexual abuse has occurred, how safety from ongoing abuse can be achieved and healing facilitated. The review found that work is needed to inform an internationally coherent conceptualisation of child sexual abuse. Further review of how service systems surrounding children are designed to work with the uncertainty of children’s early attempts to disclose their abuse is needed. Based on research that this review sourced, it is evident that service responses need to be sensitive to the process involved in children’s disclosures instead of seeing disclosure as a one-off event. Understanding disclosure processes is an opportunity to bridge the chasm between self-reports and official data on CSA prevalence and to incorporate lived experience knowledge into practice responding to this global phenomenon. The study concludes with a call for research addressing the identified priorities.

## References

[CR1] Alaggia, R., Collin-Vézina, D., & Lateef, R. (2019). Facilitators and barriers to child sexual abuse (CSA) disclosures: A research update (2000–2016). *Trauma, Violence, & Abuse,**20*(2), 260–283. 10.1177/1524838017697312 10.1177/1524838017697312 PMC6429637 29333973

[CR2] Arksey, H., & O’Malley, L. (2005). Scoping Studies: Toward a methodological framework. *International Journal of Social Research Methodology,**8*(1), 19–32. 10.1080/1364557032000119616

[CR3] Australian Bureau of Statistics (ABS). (2019). *Recorded crime—victims, Australia, 2019*. ABS cat. no. 4510.0. Canberra: ABS. https://www.abs.gov.au/statistics/people/crime-and-justice/recorded-crime-victims/latest-release#data-downloads

[CR4] Bernard, C. (2019). Using an intersectional lens to examine the child sexual exploitation of black adolescents. In *Child sexual exploitation: Why theory matters* (pp. 193–208). Policy Press. 10.51952/9781447351429.ch010

[CR5] Blunden, H., Giuntoli, G., Newton, B. J., & Katz, I. (2021). Victims/survivors’ perceptions of helpful institutional responses to incidents of institutional child sexual abuse. *Journal of child sexual abuse,**30*(1), 56–79. 10.1080/10538712.2020.1801932 33017277

[CR6] Braun, V., & Clarke, V. (2006). Using thematic analysis in psychology. *Qualitative Research in Psychology,**2*(1), 77–101.

[CR7] Braun, V., & Clarke, V. (2021). One size fits all? What counts as quality practice in (reflexive) thematic analysis?. *Qualitative Research in Psychology, 18*(3), 328–352.

[CR8] Broaddus-Shea, E. T., Scott, K., Reijinders, M., & Amin, A. (2021). A review of the literature on good practice considerations for initial health system response to child and adolescent sexual abuse. *Child Abuse & Neglect,**116*, 104225–104225. 10.1016/j.chiabu.2019.104225 31711682 10.1016/j.chiabu.2019.104225

[CR9] Cross, T. P., Walsh, W. A., Simone, M., & Jones, L. M. (2003). Prosecution of child abuse: A meta-analysis of rates of criminal justice decisions. *Trauma, Violence & Abuse,**4*(4), 323–340. 10.1177/1524838003256561 10.1177/1524838003256561 15006300

[CR10] Gorissen, M., van den Berg, C., Bijleveld, C., Ruiter, S., & Berenblum, T. (2021). Online disclosure of sexual victimisation: A systematic review. *Trauma, Violence & Abuse*. Online First. 10.1177/15248380211043831. 10.1177/15248380211043831 34634969

[CR11] Herbert, J. L., & Bromfield, L. (2016). Evidence for the efficacy of the child advocacy center model: A systematic review. *Trauma, Violence & Abuse,**17*(3), 341–357. 10.1177/1524838015585319 10.1177/1524838015585319 25971710

[CR12] Herbert, J. L., & Bromfield, L. (2019). Better together? A review of evidence for multi-disciplinary teams responding to physical and sexual child abuse. *Trauma, Violence, & Abuse,**20*(2), 214–228. 10.1177/1524838017697268 10.1177/1524838017697268 29334012

[CR13] Kennedy, A. C., & Prock, K. A. (2018). “I still feel like I am not normal”: A review of the role of stigma and stigmatisation among female survivors of child sexual abuse, sexual assault, and intimate partner violence. *Trauma, Violence & Abuse,**19*(5), 512–527. 10.1177/1524838016673601 10.1177/1524838016673601 27803311

[CR14] Khoo, E., Mancinas, S., & Skoog, V. (2015). We are not orphans. Children’s experience of everyday life in institutional care in Mexico. *Children and Youth Services Review,**59*, 1–9.

[CR15] Kim, S., Noh, D., & Kim, H. (2016). A summary of selective experimental research on psychosocial interventions for sexually abused children. *Journal of Child Sexual Abuse,**25*(5), 597–617. 10.1080/10538712.2016.1181692 27472511 10.1080/10538712.2016.1181692

[CR16] Klebanov, B., Friedman-Hauser, G., Lusky-Weisrose, E., & Katz, C. (2024). Sexual Abuse of Children With Disabilities: Key Lessons and Future Directions Based on a Scoping Review. *Trauma, Violence, & Abuse,**25*(2), 1296–1314. 10.1177/15248380231179122 10.1177/15248380231179122 37306024

[CR17] Macdonald, G., Higgins, J. P. T., Ramchandani, P., Valentine, J. C., Bronger, L. P., Klein, P., O’Daniel, R., Pickering, M., Rademaker, B., Richardson, G. & Taylor, M. (2012). Cognitive‐behavioural interventions for children who have been sexually abused. *Cochrane Database of Systematic Reviews, 5*. 10.1002/14651858.CD001930.pub3. 10.1002/14651858.CD001930.pub3 PMC7061273 22592679

[CR18] Mathews, & Collin-Vézina, D. (2019). Child Sexual Abuse: Toward a Conceptual Model and Definition. *Trauma, Violence, & Abuse,**20*(2), 131–148. 10.1177/1524838017738726 10.1177/1524838017738726 PMC6429628 29333990

[CR19] Mathews, B., Pacella, R. E., Scott, J. G., Finkelhor, D., Meinck, F., Higgins, D. J., Erskine, H. E., Thomas, H. J., Lawrence, D., Haslam, D. M., Malacova, E., & Dunne, M. P. (2023). The prevalence of child maltreatment in Australia: Findings from a national survey. *Medical Journal of Australia,**218*(6 Suppl), S13–S18. 10.5694/mja2.51873 37004184 10.5694/mja2.51873 PMC10953347

[CR20] McCalman, J. Bridge, F Whiteside, M., Bainbridge, R, Tsey, K., & Jongen, C., (2014). Responding to Indigenous Australian Sexual Assault: A Systematic Review of the Literature. *Sage Open. *https://hdl.handle.net/10018/1245067

[CR21] McTavish, J. R., Santeso, N., Amin, A., Rejnders, M., Ali, M. U., Fitzpatrick Lewisi, D., & MacMillan, H. L. (2021). Psychosocial interventions for responding to child sexual abuse: A systematic review. *Child Abuse and Neglect,**116*, 104203. 31677720 10.1016/j.chiabu.2019.104203

[CR22] Michell, D. (2015). Foster care, stigma and the sturdy, unkillable children of the very poor. *Continuum: Journal of Media & Cultural Studies,**29*(4), 663–676.

[CR23] Moher, D., Liberati, A., Tetzlaff, J., Altman, D. G., The PRISMA Group. (2009). Preferred Reporting Items for Systematic Reviews and Meta-Analyses: The PRISMA Statement. *PLoS Med,**6*(7), e1000097. 10.1371/journal.pmed1000097 19621072 10.1371/journal.pmed.1000097 PMC2707599

[CR24] Nwogu, N. N., Agrawal, L., Chambers, S., Buagas, A. B., Daniele, R. M., & Singleton, J. K. (2015). Effectiveness of child advocacy centers and the multidisciplinary team approach on prosecution rates of alleged sex offenders and satisfaction of non-offending caregivers with allegations of child sexual abuse: A systematic review. *JBI Evidence Synthesis,**13*(12), 93–129. 26767818 10.11124/jbisrir-2015-2113

[CR25] Pereda, N., Guilera, G., Forns, M., & Gomez-Benito, J. (2009). The international epidemiology of child sexual abuse: A continuation of Finkelhor (1994). *Child Abuse & Neglect,**33*, 331. 19477003 10.1016/j.chiabu.2008.07.007

[CR26] Pickering, C., & Byrne, J. (2014). The benefits of publishing systematic quantitative literature reviews for PhD candidates and other early-career researchers. *Higher Education Research & Development,**33*(3), 534–548.

[CR27] Porges, S. W. (2020). The Handbook of Therapeutic Care for Children. In J. Mitchell, J. Tucci, & E. Tronick (Eds.), *Feeling Safe is the Treatment. *Jessica Kingsley publishers.

[CR28] Reitsema, R. M., & Grietens, H. (2016). Is anybody listening? The literature on the dialogical process of child sexual abuse disclosure was reviewed. *Trauma, Violence & Abuse,**17*(3), 330–340. 10.1177/1524838015584368 10.1177/1524838015584368 25951841

[CR29] Sanchez de Ribera, O., Trajdenberg, N., & Christenson, L. (2020). Evaluating the quality of meta-analytical reviews regarding child sexual abuse interventions. *Child Abuse and Neglect,**104*, 104463. 10.1016/j.chiabu.2020.104463 32240874 10.1016/j.chiabu.2020.104463

[CR30] Satapathy, S., Choudhary, V., & Sagar, R. (2016). Tools to assess psychological trauma & its correlates in child sexual abuse: A review & current needs in Asia. *Asian Journal of Psychiatry,**25*, 60–73. 10.1016/j.ajp.2016.10.012 28262176 10.1016/j.ajp.2016.10.012

[CR31] Sawrikar, P., & Katz, I. (2017). Barriers to disclosing child sexual abuse (CSA) in ethnic minority communities: A review of the literature and implications for practice in Australia. *Children and Youth Services Review,**83*, 302–315.

[CR32] Scoglio, A. A. J., Kraus, S. W., Saczynski, J., Jooma, S., & Molnar, B. E. (2021). Systematic review of risk and protective factors for revictimisation after child sexual abuse. *Trauma, Violence, & Abuse,**22*(1), 41–53. 10.1177/1524838018823274 10.1177/1524838018823274 30669947

[CR33] The Royal Commission into Institutional Responses to Child Sexual Abuse (2017). Available at https://www.childabuseroyalcommission.gov.au/10.1016/j.chiabu.2017.09.031 29037437

[CR34] Tichelaar, H. K., Deković, M., & Endendijk, J. J. (2020). Exploring effectiveness of psychotherapy options for sexually abused children and adolescents: A systematic review of randomised controlled trials. *Children and Youth Services Review,**119*, 105519. 10.1016/j.childyouth.2020.105519

[CR35] Van Ham, K., Hoytema van Konijnenburg, E. M. M., Brilleslijper-Kater, S. N., Schepers, A., Daams, J. G., Teeuw, A. H., van Rijn, R. R., & van der Lee, J. H. (2020). A systematic review of instruments used to assess nonverbal emotional signs in children during an investigative interview for suspected sexual abuse. *Child Abuse Review,**29*(1), 12–26. 10.1002/car.2601

[CR36] Vizard, E. (2013). Practitioner Review: The victims and juvenile perpetrators of child sexual abuse - assessment and intervention. *Journal of Child Psychology and Psychiatry,**54*(5), 503–515. 10.1111/jcpp.12047 23397965 10.1111/jcpp.12047

[CR37] Voogt, A., Klettke, B., & Crossman, A. (2019). Measurement of victim credibility in child sexual assault cases: A systematic review. *Trauma, Violence, & Abuse,**20*(1), 51–66. 10.1177/1524838016683460 10.1177/1524838016683460 30803401

[CR38] Vosz, M. (2021). *Participation is not enough practices associated with giving due weight to the views of children and young people in out-of-home care policymaking in Australia*. PhD thesis. Southern Cross University. 10.25918/thesis.203.

[CR39] Wright, K., Swain, S., & McPhillips, K. (2017). The Australian royal commission into institutional responses to child sexual abuse. *Child Abuse & Neglect, 74*, 1–9. 10.1016/j.chiabu.2017.09.031 29037437

[CR40] World Health Organisation. (1999). *Report of the consultation on child abuse prevention*. Geneva, Switzerland. http://apps.who.int/iris/handle/10665/65900.

[CR41] Zaffarese-Dippold, S. M. (2017). The lived experience of former foster children who had to move their belongings in garbage bags. In *Dissertation abstracts international section A: Humanities and social sciences*. Capella University

